# Qualitative exploration of the acceptability of a postnatal pelvic floor muscle training intervention to prevent urinary incontinence

**DOI:** 10.1186/s12905-019-0878-z

**Published:** 2020-01-17

**Authors:** Aileen Grant, Sinead Currie

**Affiliations:** 1 School of Nursing and Midwifery, Robert Gordon University, Aberdeen, AB14 7QG UK; 2 Psychology, Faculty of Natural Sciences, University of Stirling, Scotland, FK9 4LA UK

**Keywords:** Urinary incontinence, Pelvic floor muscle training, Physical activity, Postnatal care

## Abstract

**Background:**

Childbirth is a major risk factor for urinary incontinence (UI). As a result, pelvic floor muscle training (PFMT) is commonly recommended during and after pregnancy to prevent the onset of UI. PFMT is often classed as a physical activity (PA) behaviour, hence PA guidelines for postnatal women encourage PFMT alongside aerobic activities. However, postnatal lifestyle interventions tend to overlook PFMT which can be detrimental to women’s health and future health risks, including urinary incontinence. This study aimed to explore perceptions and acceptability of a postnatal physical activity and PFMT intervention with postnatal women in Scotland.

**Methods:**

We recruited women who had given birth within the last 5 years by displaying posters in health centres and community centres in Stirling and through Facebook. Data was gathered via online and face-to-face focus groups, that were audio recorded and transcribed verbatim. Analytic themes were initially organised under related concepts derived from the topic guide and thematic analysis conducted. Subsequent analysis was by the Framework technique.

**Results:**

A total of seven online and face-to-face focus group discussions with 31 women identified there was a clear intention behaviour gap for engagement in PA, with both psychological and logistical barriers identified such as motivation and childcare. This was distinct from PFMT where there was a feeling of helplessness around not knowing how to perform a correct PFMT contraction subsequently resulting in women not adhering to PFMT guidance. Women felt there was no accessible PFMT advice available through the NHS. Some participants had received PFMT advice after childbirth and spoke of the Squeezee app being useful in adhering to a PFMT regimen but they did require additional teaching on how to do correct contractions. There was need for clarity and practical support for PFMT in the postnatal period with an approved intervention incorporating an accessible app being suggested by participants.

**Conclusions:**

Women would like to be trained on postnatal PFMT but face barriers to accessing adequate information and education on how to do a PFMT contraction. An intervention combining PFMT training and an app would be the most useful for their needs and circumstances.

## Background

Urinary incontinence (UI, defined as the involuntary leakage of urine) is a common problem among women, affecting between 25 and 45% internationally [1]. There are different types of UI with stress incontinence (involuntary leakage from coughing, sneezing, effort or exertion) being the most common affecting approximately 10–39% of women [2, 3]. The cost to the United Kingdom’s National Health Service (NHS) was estimated at £233million in 2000 [4] and the personal costs to women estimated to be £178 million [5]. UI symptoms affect quality of life [6], sexual function, and prevent engagement with fitness and exercise activities [7, 8] and are associated with major depression [9], social isolation and psychological distress in older women [10]. Pelvic floor muscle training (PFMT) is the first line treatment for stress UI [11] and there is good evidence to show PFMT can cure or improve symptoms [12]. There is also evidence PFMT interventions can improve knowledge and skills about PFMT and UI [13] and PFMT can prevent UI [7, 14] which suggests it should be a routine part of women’s exercise activities.

Childbirth is a major risk factor for UI due to stretching and damage to the pelvic floor [7]. As a result, PFMT is commonly recommended during pregnancy to prevent the onset of UI [15]. However, women are bombarded with health messages during their antenatal care, with PFMT often not recognised as a priority and without adequate support women feel disempowered which leads to a lack of self-efficacy and engagement [16]. Age, pregnancy, childbirth and an increased body mass index are all risk factors for urinary incontinence [3, 17, 18] suggesting there is opportunity for postnatal intervention to aim to prevent and/or treat UI. Nonetheless, there is uncertainty whether a postnatal population-based approach for delivering PFMT is effective or acceptable in preventing or reducing urinary incontinence in the long-term [14]. Chiarelli and Cockburn’s trial evaluating the effectiveness of a physiotherapist delivered intervention designed to prevent UI in postnatal women showed the prevalence and severity of UI was reduced 3 months later [19]. Nevertheless, this trial was published in 2002 and there is a need for longer term improvement to address the costs to the NHS and women suffering from UI. A more recent review by Morkved and Bo has shown the evidence in this area is mixed with some studies showing statistically significant effects and other which do not [7]. Variation in the characteristics of women included, content of interventions and outcome measures used are likely to contribute to these variations. Therefore, more supporting evidence and appropriately designed interventions which reflect and acknowledge technological advances are required.

International guidelines, including those from the UK, encourage women to engage in regular physical activity. In the UK, guidelines recommend 150 min of moderate to vigorous intensity physical activity per week [20], with postnatal women recommended to gradually work towards this target around four to 6 weeks post birth [21]. These guidelines tend to encourage aerobic activities, strengthening, stretching and walking but also include pelvic floor exercises [21]. Adherence to these guidelines is associated with a range of health benefits such as improved psychological wellbeing, improved cardiovascular fitness and weight management [22, 23]. However, many women find it challenging to be physically active and meet guidelines after having a baby. Research indicates that fatigue, motivation, confidence and lack of resources are key barriers to regular physical activity (PA) [24].

There are a range of postnatal interventions that aim to improve physical activity in this population and tackle barriers. However, many of these interventions are weight management focused with more emphasis on clinical outcomes rather than physical activity behaviour change [25]. Indeed, interventions which have a focus on physical activity behaviour with key behaviour change techniques such as goal setting and self-monitoring, are seen to be more effective at improving physical activity compared with interventions which focus on clinical outcomes such as weight loss [25]. Although postnatal PA guidelines often include advice to perform pelvic floor muscle training, postnatal lifestyle interventions tend to overlook this activity and do not address or recognise it as a physical activity behaviour, focusing instead on aerobic or cardiovascular based physical activity behaviours. This can be detrimental to women’s health as PA including PFMT is required in order to improve physical and mental health as well as reduce future health risks, such as urinary incontinence.

This paper presents the findings from an exploratory qualitative study with postnatal women in Scotland that aimed to explore perceptions and experience of postnatal PA and PFMT and explore the acceptability of a postnatal physical activity and PFMT intervention.

## Methods

This qualitative exploratory study utilised focus group discussions to explore perceptions and experiences, and barriers and facilitators of postnatal physical activity (including PFMT), and the acceptability of support to enable physical activity and PFMT. Women were eligible to participate if they were 18 years of age or older and had given birth to one or more children in the last 5 years. Women were offered a £5 shopping voucher as an incentive.

Participants were recruited via posters displayed in community centres, libraries and other public spaces and Facebook. On Facebook a group page was created to advertise the study to as wide an audience as possible and maximise the variation in our sample. The page was used as a platform to maintain engagement with interested parties through regular updates of when and where focus groups were taking place.

Focus groups were guided by a topic guide (informed by the literature) and data were collected in three different ways: face-to-face, online audio/visual and online written (topic guide is available as an Additional file 1). To aid engagement from participants online audio/visual discussions were conducted using video conferencing software (Zoom) and written discussions took place through the Facebook private ‘chat room’ page [26]. Face-to-face focus groups took place at the University of Stirling and in community centres and lasted approximately 45 to 60 min. The face-to-face and online audio/visual focus groups were audio recorded and transcribed verbatim. Transcripts were anonymised through the use of pseudonyms and imported into Nvivo 11.

Transcripts were read and re-read and an initial coding frame developed. This was then systematically applied to all transcripts within Nvivo 11. Themes were initially organised under related concepts derived from the topic guide and thematic analysis. Subsequent analysis was by the Framework technique [27]. Thematic charting facilitated comparing the data by concept, theme and group. The data were explored for negative cases which helped construct a more nuanced explanation.

This study was approved by the University of Stirling, General University Ethics Panel (GUEP105).

## Results

A total of 31 women took part in seven focus groups (three face-to-face, three online audio/visual, and one online written) which took place between May and August 2018. The age of participants ranged from 28 to 43 and participants either had one or two children and these children varied in age between 7 months and 5 years. The majority of participants were married [9] with the remainder cohabiting or single. The majority of participants were educated to degree or masters level [9] with the remainder reporting a professional qualification, no qualifications or declining to comment. The findings from this study are reported under three themes: perceptions and experiences of engagement in postnatal physical activity, perceptions and experiences of postnatal pelvic floor muscle training and potential postnatal intervention features.

### Perceptions and experiences of engagement in postnatal physical activity

All participants reported they wanted to be physically active on a regular basis but the majority reported not engaging in physical activity at the time of the focus groups. Throughout all focus groups time was consistently identified as the biggest barrier to physical activity.

“*Time is a huge thing obviously … you just don’t have the same time.”* (FG 2, P1)

And

*“I think time is a big barrier to me at the moment … working it all around bed times and feeding and all that kind of stuff, it’s a bit tricky.”* (FG3, P2)

Women also mentioned fatigue, physical pain (including arthritis and back pain), prolapse, body image and confidence as barriers to engagement in physical activity.

*“I don’t want to go to the gym because I’m embarrassed about the size I am; I’ve not really got the confidence to go there.”* (FG3, P1)

The most frequently reported facilitators of engagement in physical activity were the psychological benefits of improving mental health and having “me-time” and the impact of physical activity on weight loss.

*“ … my biggest motivation is just wanting to be more toned and get back to normal again.”* (FG 3, P2)

Others reported wanting to set a good example to their children:

*“I want my kids to be active, and I think the best way to show them that, is to be active myself … so it becomes that habit for them as well.”* (FG 5, P4)

Participants reported variation in the availability of local physical activity facilities which accommodate children. Some reported buggy exercise activities in their local park but others reported that the information about groups on the internet is not up-to-date and many groups are not running anymore. Others reported the availability of crèche facilities in local gyms, walking and pushing the buggy and using relatives to babysit enabled them to engage in physical activity. The barriers reported were closely associated, such as, husband working away, not living near family, having less money for gym membership and crèche facilities whilst being on maternity leave or working part-time. Participants who reported engaging in physical activity before they were pregnant reported less barriers to engaging in physical activity.

*“I did kind of more extreme sports, quite a lot of sailing and skiing. I quite like going for a run or a really brisk walk. I go out with the buggy as much as I can and I’ve been doing a fit camp … I’m hoping I can kind of gradually ramp it up, because I’d like to be (doing more) as time goes on.”* (FG 7, P8)

Conversely, women who had never been physically active displayed lower levels of intention to become active in the future. One participant declared:

*“I find it very difficult to do any physical activity but I was the same before having my child as well … it’s not something that I’ve been interested in ore something I’ve been motivated to do, it’s a chore.”* (FG 1, P1)

### Perceptions and experiences of postnatal pelvic floor muscle training

All but one participant was aware they should be doing PFMT after having a baby. Most of these participants reported they were told they should engage in PFMT once they were pregnant. This was usually by their midwife who told them they should be doing PFMT and provided a leaflet. One participant reported not being told by their midwife to do PFMT so was not aware she should be doing PFMT until after childbirth:

*“I don’t think I did them when I was pregnant, I don’t think I really knew about them. I don’t remember ever thinking about it or it wasn’t until I think they came to see me after I’d had my baby to tell me that I should be doing pelvic floor exercises and I got a leaflet. I think that was maybe the first time I’d ever heard about them.”* (FG 3, P2)

Participants who received a leaflet were never taught the correct PFMT contraction and some found this a barrier to engaging and maintaining PFMT.

*“I was given a leaflet, ehm nobody really demonstrated or kind of showed you how to do the pelvic floor exercises.”* (FG 3, P4)

And

*“I find it a very difficult exercise to know how to do, I tend to squeeze my stomach in and think “oh i’m not doing it right?”” “No-one can really watch you doing it, so you kinda presume you’re doing it right*.” (FG 1, P1 and P2)

Some participants reported receiving a physiotherapy support after the birth of their child. This assessment involved checking their abdominal muscles and ensuring they knew how to do a correct PFMT contraction.

*“I didn’t get any support after I had (first child) with pelvic floor or even any discussion. Then when we moved to West Lothian, and then I had (child named), and West Lothian do a, they come round, the physios when you’re in your bed after you’ve had the baby and talk about pelvic floor and go through a sheet of exercises with you so I went home with that, so I think that was really helpful … ”* (FG 2, P3)

However, one of the participants who was offered a post-natal physio service described her son being too ill for her to receive this service.

*“ … in Glasgow a physio sees you after, sees everybody in the postnatal ward. So a physio came to see me, checked my abs and checked that I knew how to do pelvic floor but because my son was so ill she was like ‘oh you don’t need to be worrying about this right now.”* (FG 2, P5)

Another participant reported having a prolapse after childbirth and paying privately to access appropriate care:

*“ … I didn’t really get any advice or anything like that, and after I had (baby named) I had huge pelvic floor issues, I ended up with a, ehm mild prolapse after having (baby named), so I really could’ve done with support and I had actually went to my GP and said ‘look something’s not right, I need a referral to a physio or something’ and she said ‘oh we don’t do that’ so I ended up seeing someone private to get advice and everything.”* (FG 4, P1)

All participants felt that the benefits of PFMT should be more widely discussed in society, specifically with women before they have children, and that information should be more readily available. They felt the benefits of PFMT were not widely discussed because of taboo issues:

*“It’s something that a bit of a weird taboo in society, speaking about women’s private parts and it’s a shame, because if women talk about bladder problems and what happens early on. Then maybe you’d do something to stop that from happening. Whereas, it’s only kind of mentioned to you when you start having babies … ”* (FG 1, P2)

### Potential postnatal intervention features

There was a consensus view that there is a lack of aftercare for women after childbirth and in particular, with PFMT.

*“I think if the health visitor had more sign posting information about, like, keeping yourself well and, like, how to get you back to being physically active and things, that would’ve helped … the health visitor did focus primarily on the health of the baby and your mental health...”* (FG 3, P3)

All participants expressed that an intervention would be a good idea and there was consensus around potential features:

### Physiotherapist session to teach correct PFMT contraction

There was consensus that generally women do not know how to do a pelvic floor contraction and therefore, were unsure if they were doing a correct contraction when they tried to engage in PFMT. Some participants suggested a physiotherapist check should replace the GP check at 6 weeks post-birth, as they felt a recommendation and sign posting to physical activity and teaching a PFMT contraction would be better than the current GP service.

*“...I saw a GP who did nothing, you know that (a physiotherapist) would be far more beneficial just to check, I dunno like your muscles are healing and everything correctly and have some sort of antenatal maternity check. I feel like they totally didn’t have anything at all.”* (FG 2, P1)

And another participant paid privately for this service:

*“...with both of them, after I got to the point of being able to exercise, I went for a full postnatal check-up with the physio, cos I think like when, you know, you’re worried about your back, I think that gave me reassurance that everything was okay to exercise and that back problems are normal until you build up your core, ehm, I think that helped me be more confident in doing it and not so worried that something was going to happen to me.”* (FG 3, P4)

And

*“P2 Yeah, they kind of focus on the wrong thing. Like I felt like every appointment you went to they talked to you about contraception. You've all been using contraception for a lot of years.*

*P6 Yeah. They're kind of like lecturing you about something you already know about. And then they're not telling you about things that you do need to get educated on, that you haven't really caught onto yet.”* (FG 4)

Some participants felt 6 weeks was too soon for a health check:

*“P3: The check-up is quite soon after you have a baby isn’t it, you’re just ‘Oh I survived, I’m alright, I’m looking after this baby, I think ok,’ at six weeks you’re not really thinking about yourself and getting back into fitness yet are you? But at 12 weeks you probably are more thinking of that.*

*P8: Yeah 12 weeks would definitely be more realistic for getting your head round, cos yeah six weeks in you aren’t back to thinking about your own body yet. You’re just still just coping, just getting through, very much still living day-to-day at that stage.”* (FG 3)

Others felt 6 to 8 weeks was an appropriate time

*“I think maybe six/eight weeks after is maybe a good time”.* (FG 6, P3)

### Support

There was a general feeling that incontinence is taboo and by having support women would be more likely to talk about it and feel less isolated. Some participants reported not being aware of the prevalence of incontinence and prolapse after childbirth until they experienced it themselves. As one participant explains:

*"I was quite down when I had my son about what was going on. And I thought that it would only sort of happen to very few people. But it was only after that and going to the GP that you realise how common it is."* (FG 1, P1)

### App

All participants felt a free NHS approved or branded app would be very useful. The participants explained that everyone has a smartphone and generally has them quite close by. In three focus groups there was at least one participant who had experience of the squeezee app. (This is an app recommended by physiotherapists to remind people to do their PFMT contractions. Women can set the app to remind them to do PFMT at convenient times of day and modify their regimen as their muscles become stronger. It is not free and participants paid £3.99).

*“ … you spend time on your phone and it’s the reminder that reminds me, And it teaches you how to do them, it kind of gives you step by step what to do it times you records you’ve done it … .Its great... Yeah like the long hold you’re supposed to do if you can’t do it for ten seconds you can do it for five or whatever and build it up so you can change it you can change it”* (FG 2)

And

*“ … because you always have your phone on you essentially, most people have some sort of electronic device on them pretty much at all times even at you’re not actively using it at the time, it’s still in your pocket.”* (FG 5)

Other participants had not heard of the app and incorrectly assumed it taught a correct contraction however upon learning it only reminded women to do their PFMT exercises felt the app would still be useful if they could also be taught a correct PFMT contraction. There was consensus about the features of an app to support PFMT, suggesting features such as: explaining why PFMT is important, the benefits of maintaining a regimen, diagrams of the muscles involved and recording of PFMT regimen so progress can be monitored. As one participant explained this would allow women to compete against themselves:

*“I quite enjoy being quite competitive with myself and trying to achieve wee goals and being on my own with exercise.”* (FG 3)

## Discussion

This study explored perceptions and experiences of postnatal physical activity and PFMT. The participants of this study did not have concerns about what physical activity they could do but most had an intention behaviour gap of actually doing physical activity. Participants reported a number of barriers to engaging in physical activity, some of which were child specific. The participants also reported a number of facilitates which could enable women to engage in physical activity if they had access to the required resources. Those who were active before pregnancy were more likely to be able to overcome barriers than those who were less active before pregnancy. The reported barriers to postnatal physical activity were common and similar to those expressed by other postnatal populations [24]. There are clearly both psychological and logistical barriers to engagement in PA behaviours which should be components of future postnatal PA interventions. Interestingly, participants implicitly indicated a clear distinction between the barriers and facilitators to general physical activity (aerobic or cardiovascular based activities) and PFMT. Hence any future intervention aiming to address these two areas of physical activity should consider their differing determinants.

In contrast, there was less understanding and more ‘helplessness’ around PFMT. Before pregnancy and childbirth most participants were not aware of the importance of PFMT or the consequences of not engaging in PFMT. Once pregnant or after childbirth participants were primarily given leaflets and told to do PFMT but participants reported a lack of certainty as to how to do a correct PFMT contraction as a barrier to maintaining PFMT regularly. Participants experienced barriers to obtaining training in PFMT through the NHS. Some women received PFMT training immediately after childbirth but there was a consensus that this was not an appropriate time to be able to retain the information given. All participants felt there was a need for a postnatal intervention which particularly focuses on PFMT to prevent the long-term problems of not engaging in PFMT such as incontinence and prolapse. The participants were in agreement this intervention should provide PFMT training, an app with information and reminders to do PFMT regularly and signposting to further information. We have not identified another study which has explicitly explored women’s perceptions and views on the acceptability of a post-natal intervention specifically addressing PFMT and physical activity. Rosqvist et al. evaluated the feasibility and acceptability of a PFMT and bladder training intervention for the treatment of UI and concluded their predefined intervention was acceptable and feasible [28]. Asklund et al. found an app based treatment program for urinary incontinence empowered women and enabled them to self-manage their incontinence treatment [29].

The lack of studies exploring the acceptability and design of postnatal PFMT interventions may be due to a more recent move within the health research community towards establishing acceptability before commencing a body of work. Much of the published research in this area is centred on evaluating the effectiveness of interventions rather than upon developing interventions. Research has focused on the efficacy of treatment to cure or improve UI symptoms once they have manifested [14, 30–32]. The preventative efficacy of PFMT is less well known, although evidence does suggest it is effective when training is conducted [7, 15]. There is a need for more research into postnatal PFMT [7]. A systematic review has shown post-natal PFMT can prevent and treat urinary incontinence but the optimum dose and duration for effective PFMT is unknown [7]. The authors recommended a training protocol for at least an 8 week training period, which was not discussed in our focus groups but is an important consideration in further work. Given the detrimental effect of not engaging in PFMT there is a need for guidelines and supporting interventions [7].

A strength of this study is that it appears to be the first to explicitly explore perceptions and acceptability before designing and evaluating a PFMT intervention. An advantage is the use of online focus groups which enabled women with childcare responsibilities to participate and to obtain perspectives and experiences of women from different locations in Scotland. The sampling method had the disadvantage of using a combination of Facebook and posters which limited the geographical locations from which we could recruit. Also participants were self-identifying so their views may not be representative of the whole population. Furthermore, we recognise using a £5 shopping voucher may have introduced bias to our sample. Given the number of women involved in this study there is a need for further work in a larger population to validate these findings.

## Conclusions

Women face barriers to accessing adequate information and education on how to do a correct PFMT contraction and this prevents them engaging and maintaining a PFMT regime. This research illustrates women would like to be educated and trained on PFMT during the postnatal period, ideally between 6 and 12 weeks after giving birth. The participants in this study suggested an intervention combining PFMT training and app would be the most useful for their needs and circumstances. Although we have focused on UI it is likely such an intervention would also prevent prolapse, other types of incontinence and improve sexual function after childbirth. There is little research exploring the acceptability of postnatal PFMT and PA interventions and this appears to be the first study to explicitly explore perceptions and experiences. This study lays good foundations for further research to design an efficacious treatment and/or prevention postnatal PFMT and PA intervention.

## Supplementary information

**Additional file 1.** Topic guide.

## Data Availability

The dataset generated and/or analysed during the current study are available from the corresponding author on reasonable request. This is sensitive data where participants have at times shared their personal experiences of incontinence and prolapse and given this we believe it would be insensitive to make the data publically available without explicit consent for this purpose.

## Abbreviations

NHS: National Health Service
PA: Physical activity
PFMT: Pelvic floor muscle training
UI: Urinary incontinence
