# Supplementary material for: Qualitative exploration of the acceptability of a postnatal pelvic floor muscle training intervention to prevent urinary incontinence
Source: BMC Womens Health. 2020 Jan 17;20:9. doi: 10.1186/s12905-019-0878-z (PMC6967084; doi:10.1186/s12905-019-0878-z)
Supplement: Supplementary file 1 — Additional file 1. Topic guide. [file 12905_2019_878_MOESM1_ESM.docx]

**Focus group topic guide – postnatal women**

We’ll start off with physical activity and can think about/discuss the other health behaviours as we go on

Firstly, do you like doing physical exercise? *What PA did you do before you were pregnant? Did this change when you became pregnant? Why?*

Do you do any physical activity? If so, what? *Have you engaged in any physical activity since giving birth? Is this different to before children? Would you like to do more or less? Has this changed over time?*

What the barriers and facilitators you face in doing physical activity? *(explore prolapse, incontinence, PND, etc)*

Do you currently do any pelvic floor muscle training? Have you ever done any PFMT?

What the barriers and facilitators you face in doing PFMT?

What do you think of the current support and information for post-natal women about physical activity, including pelvic floor muscle training?

Is there any other healthy behaviours you would like to take part in?

What are the barriers and facilitators to these?

What do you think of the current support and information for post-natal women about these health behaviours?

Do you think this information could be improved? Is there any information missing? If so, how do you think this could be improved? What format should it take?

Would you like support? If so, how?

Do you have any comments or any information you would like to share with us which we have not covered?

**End of questions about healthy behaviours. We will ask women if they would mind commenting for a few extra minutes about the research methodology they have engaged with.**

Why did you use this method?

Have you enjoyed your experience?

Is there anything you liked or disliked about it?

Would you take part in a study using the format again?

Were you interested in taking part in any of the other formats? Why didn’t you?
